# Antiretroviral Regimens and CD4/CD8 Ratio Normalization in HIV-Infected Patients during the Initial Year of Treatment: A Cohort Study

**DOI:** 10.1371/journal.pone.0140519

**Published:** 2015-10-20

**Authors:** F. De Salvador-Guillouët, C. Sakarovitch, J. Durant, K. Risso, E. Demonchy, P. M. Roger, E. Fontas

**Affiliations:** 1 Department of Infectiology, Nice University Hospital, Nice, F-06003, France; 2 Department of Clinical research and Innovation, Nice University Hospital, Nice, F-06003, France; Jackson Laboratory, UNITED STATES

## Abstract

**Background:**

As CD4/CD8 ratio inversion has been associated with non-AIDS morbidity and mortality, predictors of ratio normalization after cART need to be studied. Here, we aimed to investigate the association of antiretroviral regimens with CD4/CD8 ratio normalization within an observational cohort.

**Methods:**

We selected, from a French cohort at the Nice University Hospital, HIV-1 positive treatment-naive patients who initiated cART between 2000 and 2011 with a CD4/CD8 ratio <1. Association between cART and ratio normalization (>1) in the first year was assessed using multivariate logistic regression models. Specific association with INSTI-containing regimens was examined.

**Results:**

567 patients were included in the analyses; the median CD4/CD8 ratio was 0.36. Respectively, 52.9%, 29.6% and 10.4% initiated a PI-based, NNRTI-based or NRTI-based cART regimens. About 8% of the population started an INSTI-containing regimen. 62 (10.9%) patients achieved a CD4/CD8 ratio ≥1 (N group). cART regimen was not associated with normalization when coded as PI-, NNRTI- or NRTI-based regimen. However, when considering INSTI-containing regimens alone, there was a strong association with normalization [OR, 7.67 (2.54–23.2)].

**Conclusions:**

Our findings suggest an association between initiation of an INSTI-containing regimen and CD4/CD8 ratio normalization at one year in naïve patients. Should it be confirmed in a larger population, it would be another argument for their use as first-line regimen as it is recommended in the recent update of the “Guidelines for the Use of Antiretroviral Agents in HIV-1-Infected Adults and Adolescents”.

## Introduction

In the current era of effective combination antiretroviral regimens (cART), optimal immune restoration has become the primary goal. Monitoring the absolute CD4 lymphocyte count to maintain a level above 500/mm^3^, remains most paramount importance. In parallel, the deleterious effect of activated cytotoxic CD8 cells has been recognized [1–3], leading to persistent inflammation [4, 5], metabolic disorders [6], cardiovascular risk and premature aging [5]. Thanks to continuous improvements in the safety and efficacy of current antiretroviral combinations, clinicians can now focus on achieving a steady and effective reduction in immune activation factors and particularly activated CD8 cells.

According to recent studies [5, 7–10], the CD4/CD8 ratio is strongly associated with immunoactivation and immunosenescence in HIV-positive patients. Further, a lower or inverted (<1) ratio has been independently associated with non-AIDS defining events (nADE), mortality and markers of age-associated diseases [9, 11–13]. Interestingly, this association has been also reported in a subset of patients virologically suppressed with high CD4 cell count [9]. As a result, given its association with morbidity and mortality, the CD4/CD8 ratio appears to be a simple and reliable surrogate marker for ART efficacy in addition of CD4 cell count and viral load, including in patients with apparent immune recovery.

Furthermore, if data accumulate about factors associated with CD4/CD8 ratio normalization, little is known about the specific impact of ART regimens and results remain controversial. In an observational cohort study no association was found between treatment (Protease Inhibitor (PI)- or Non-Nucleoside Reverse Transcriptase Inhibitor (NNRTI)-based) and normalization [14] whereas Mussini et al. found that patients treated with AZT/3TC and ddI/D4T Nucleoside Reverse Transcriptase Inhibitor (NRTI) associations were less likely to normalize their ratio compare to Tdf/FTC association as a reference [13]. Furthermore, the question arise of the specific association of INtegrase Strand Transfer Inhibitor (INSTI) containing ART regimens with ratio normalization as the principal agent of this class, raltegravir, has been associated, in switch or intensification studies, with inflammation and immune activation improvement [15–21], or, directly with CD4/CD8 ratio increase in two small studies where patients were switched, or intensified, from NNRTI- or PI-based regimens to raltegravir [22, 23].

Our aim was to investigate the association of antiretroviral regimens with CD4/CD8 ratio normalization within the Nice HIV patient cohort which is part of the multicenter NADIS^®^ [24] observational cohort.

## Materials and Methods

### Data source

The Nice HIV cohort contains data on 2885 patients followed at Nice University hospital. The usual follow-up schedule consists in a consultation every 3 months. At each consultation, clinical, laboratory and treatment data are entered in real time by the physician in a computerised medical record (NADIS^®^) from which our data were retrieved. Quality control is conducted by a clinical research assistant. All patients provide written informed consent upon inclusion within the database for their data to be processed anonymously for research purposes and the database has been registered with the regulatory authority according to national regulations regarding patient consent and ethical review. The NADIS^®^ database has been approved by the French Committee on Informatics and Freedom (CNIL).

### Patient selection

We selected HIV-1 positive treatment-naive patients who initiated a first line cART between 01/01/2000 and 31/12/2011. Inclusion criteria comprised documented baseline CD4 and CD8 T-cell counts (or counts obtained within the previous 6 months) and at least another count within the following year. Patients whose baseline CD4/CD8 ratio exceeded 1 were not included. Patients were followed until the last CD4/CD8 ratio available before death, loss to follow-up or 1 year of follow-up whichever occurred first. cART changes were allowed and patients were analyzed according to their first line regimen in an intention-to-treat fashion.

### Study variables

The study outcome was the normalization of the CD4/CD8 ratio, defined as at least one ratio measurement above 1 during the year following treatment initiation. Our main independent variable, cART, was defined as a combination of 3 or more active drugs including boosted PI, NRTI, NNRTI, INSTI and/or an anti-CCR5 drug (entry inhibitors, EI, including fusion inhibitors).We considered as covariables in our models the factors known to be associated with the CD4/CD8 ratio [5, 8, 9, 14, 25–27] or potentially influencing the cART use such as calendar year. Age, BMI, baseline CD4 and CD8 T-cell counts and nadir CD4, delay between diagnosis and treatment initiation were considered as continuous variables, gender, HCV co-infection and AIDS-defining condition as binary variables, mode of transmission (Homo-bisexual, Heterosexual, Intravenous drug user (IVDU) and Other), and baseline viral load (VL, < = 500, 500–30000, >30000), and calendar year (early cART ≤2005, late cART >2005) as categorical.

We also studied dyslipidaemia as potential risk factor defined according to the thresholds established by the National Cholesterol Education program (ATP III guidelines on cholesterol): total cholesterol (TC) ≥ 6.2mmol./l, HDL cholesterol (HDL-c) ≤ 0.9 mmol/l, and triglycerides (TG) ≥ 2.3 mmol/l. Dyslipidaemia was present if at least one of these parameters was above the indicated threshold.

### Statistical analysis

Demographic and clinical characteristics at baseline were summarized with means and standard deviations (SD), medians and interquartile ranges (IQR) or frequencies and percents. We present data for the total population and for the patients who achieved CD4/CD8 ratio normalization (“Normalized” group, N) or not (“Non-Normalized” group, NN).

We fitted two different univariate logistic regression models to study the association between CD4/CD8 ratio normalization and cART coding the treatment in two different ways. First as a categorical variable: IP + NRTI (eventually combined with INSTI or EI) being the reference, NNRTI + NRTI (eventually combined with INSTI or EI), NRTI (eventually combined with INSTI or EI) and other combinations. Second, cART was coded as binary variable, INSTI-containing regimens versus non INSTI-containing regimens.

Then, in multivariate analyses, we adjusted these models on known CD4/CD8 ratio determinants described above. Dyslipidaemia, as a studied potential risk factor, was included in the models if it had a p-value less than 0.10 in univariate analysis. Collinearity was considered for covariates (e.g. for nadir CD4 and CD4 count) before entry in the models. Lastly, a sensitivity analysis has been performed considering a lower cut-off, 0.8, for ratio normalization.

All tests were two sided with a 5% level of significance. Analysis was performed using SAS, version 9.1.3 (SAS Institute, Inc., Cary, NC).

## Results

**Fig 1:**
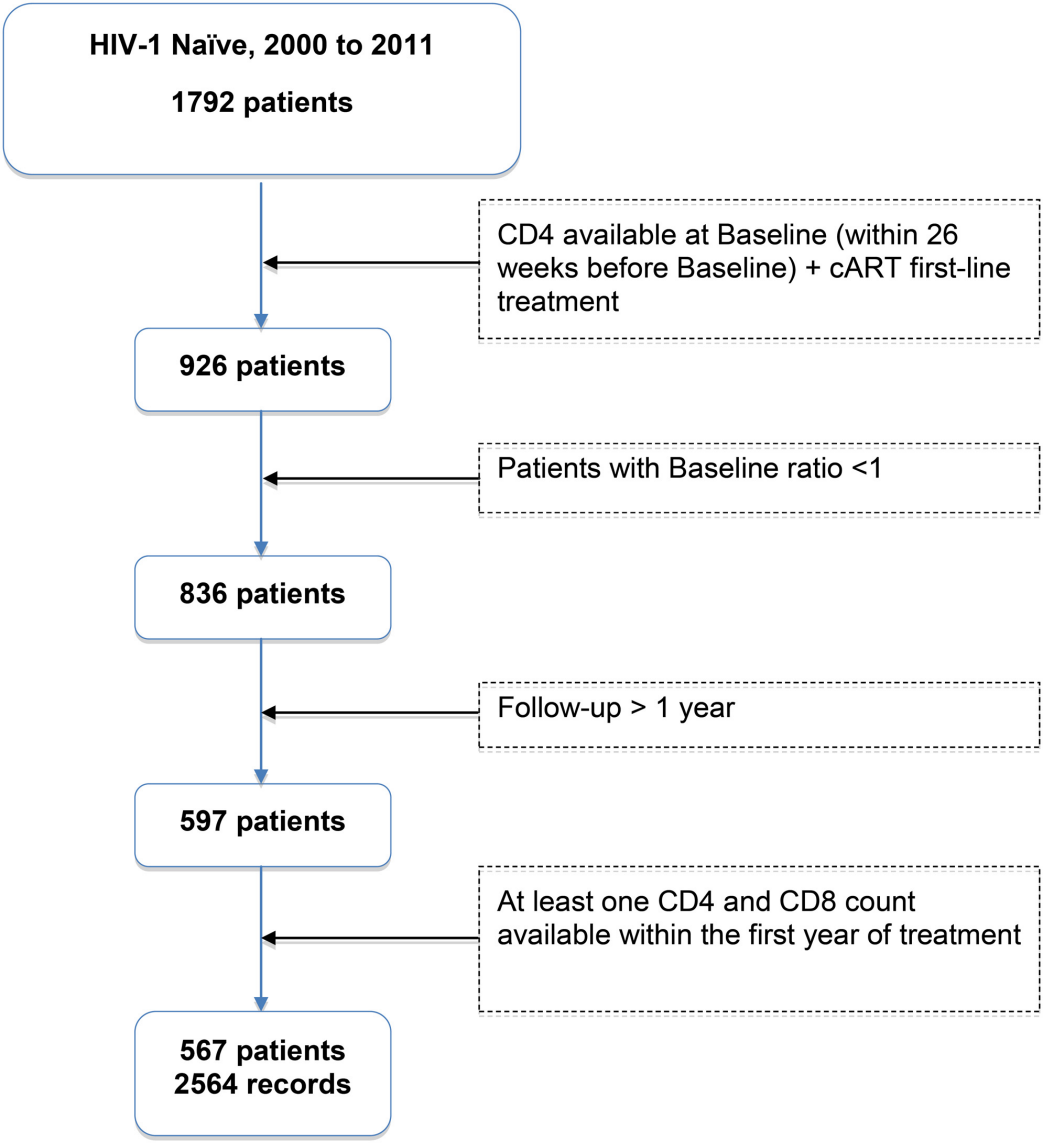
Selection of study participants.

Among the 1,792 HIV-1 infected patients included in the Nadis^®^ database between 2000 and 2011, 567 were eligible for the study (Fig 1).

At baseline, mean age was 42.5 (±10.3) years, 67.9% were men, most were contaminated through sexual transmission (heterosexual 251[44.3%]; homo- and bi-sexual 179 [31.6%]) and 87 (15.3%) through IV drug use. Median CD4 T cell count was 304 (171–476)/mm^3^, median viral load was 3.26(log10) (1.60–4.75) copies/ml and 203(40.6%) patients had undetectable (<400) viral load, 141 (24.9%) had an AIDS-defining condition and 143 (25.2%) were HCV co-infected (Table 1). Dyslipidaemia was present in 67 patients, representing 35.4% of the population with available data.

**Table 1:** Baseline characteristics of the study population.

	Total(N = 567)(100%)	NormalizedN = 62(10.9%)	Non NormalizedN = 505(89.1%)
**Age (year)**, mean ± sd	42.5±10.3	40.4±11.4	42.7±10.1
**Gender**			
Male	385	39 (10.1%)	346 (89.9%)
Female	182	23 (12.6%)	159 (87.4%)
**BMI**			
**(**kg/m^2^ )*, mean ± sd	22.7±4.1	22.2±3.4	22.8±4.2
**HCV coinfection**			
No	424	47 (11.1%)	377 (88.9%)
Yes	143	15 (10.5%)	128 (89.5%)
**AIDS-defining condition**			
No	426	55 (12.9%)	371 (88.9%)
Yes	141	7 (5.0%)	134 (95.0%)
**Mode of contamination**			
Heterosexual	251	34 (13.6%)	217 (86.4%)
Homo-bisexual	179	18 (10.1%)	161 (89.9%)
IV drug user	87	5 (5.7%)	82 (94.3%)
Other	50	6 (12.0%)	44 (88.0%)
**Calendar year**			
Early cART, ≤ 2005	134	14(10.4%)	120 (89.6%)
Late cART, > 2005	433	48(11.1%)	385 (88.9%)
**Diagnosis-to-treatment delay** (years)(mean±SD)	8.1±7.4	6.1±7.5	8.3±7.4
**Baseline CD4 count** (cells/mm^3^)(median [IQR])	304[171;476]	460[313;621]	286[155;462]
**Nadir CD4** (cells/mm^3^)(median [IQR])	277[151;427]	416[308;571]	260[138;405]
**Baseline CD8 count** (cells/mm^3^)(median [IQR])	863[602;1202]	758[480;1003]	876[610;1242]
**Baseline ratio** (median [IQR])	0.36[0.19;0.56]	0.74[0.44;0.84]	0.32[0.18;0.50]
**Viral load** (log10)* (median [IQR])	3.26[1.60;4.75]	3.26[1.60;5.00]	3.26[1.60;4.72]
Dyslipidemia*			
No	122	21 (17.2%)	101 (82.8%)
Yes	67	3 (4.5%)	64 (95.5%)
Missing	378	38 (10.0%)	340 (90.0%)
**cART treatment**			
PI-based regimens	300	36 (12.0%)	264 (88.0%)
NNRTI-based regimens	168	17(10.1%)	151 (89.9%)
NRTI-based regimens (≥3 NRTI)	59	3 (5.1%)	56 (94.9%)
Other (incl PI + NNRTI)	40	6 (15.0%)	34 (85.0%)
**INSTI-containing regimens**			
No	522	52 (10.0%)	470 (90.0%)
Yes	45	10 (22.2%)	35 (77.8%)
*incl*.			
*PI-based association*	*11*	4 (36.4%)	7 (63.6%)
*NNRTI-based association*	*3*	0 (0.0%)	3 (100.0%)
*NRTI-based association (2 NRTI)*	*25*	6 (24.0%)	19 (76.0%)
*Other (incl PI + NNRTI)*	*6*	0 (0.0%)	6 (100.0%)

Abbreviations—cART, combined antiretroviral therapy; sd, standard deviation; IQR, interquartile range. incl, including.

At baseline, 52.9%, 29.6% and 10.4% of the study population initiated respectively a PI-based, NNRTI-based or an NRTI-based cART regimen. About 8% of the patients started an INSTI-containing regimen. All the patients treated with the INSTI antiretroviral class received raltegravir.

**Fig 2:**
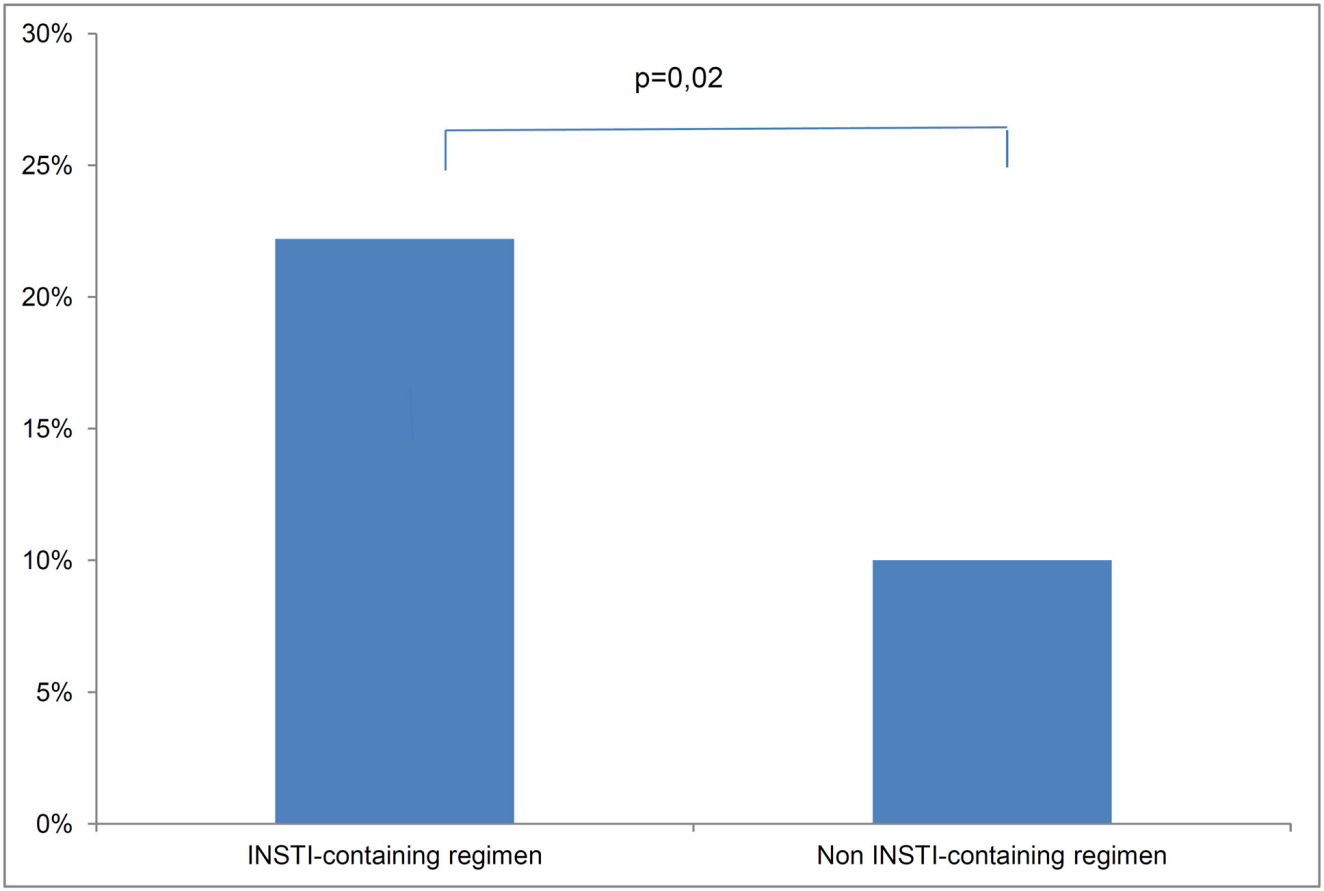
Rate of ratio normalization in INSTI- or Non INSTI-containing regimens.

Within the year following treatment initiation, 62 (10.9%) patients achieved a CD4/CD8 ratio ≥1 (N group) while 505 (89.1%) patients did not normalize their CD4/CD8 ratio (NN group). A total of 10 patients (22.2%) of the patients initiating an INSTI containing regimen normalized their ratio (Fig 2). Within the N group, 16(25.8%), 13(21.0%) and 33(53.2%) patients normalized their ratio respectively in the 3 first months, between M3 and M6, and after M6. Only 52(9.2%) patients did not normalize before M6 and had no further CD4 and CD8 measurements for evaluation. Baseline characteristics of both groups are summarized in Table 1.

In univariate analysis (Table 2), a higher baseline CD4 count, nadir CD4 count and baseline CD4/CD8 ratio were significantly associated with an increased probability of ratio normalization. Conversely, a pre-existing AIDS-defining condition or dyslipidaemia at baseline, or a higher diagnosis-to-treatment delay were associated with an increased risk of non-normalization of the CD4/CD8 ratio in the subsequent year. When coded as class-based regimen the cART treatment was not associated with the ratio normalization. However, when considering the cART treatment as a binary variable, INSTI-containing regimen or not, the association was statistically significant [OR, 2.58 (1.21–5.52)] in favour of the INSTI-containing regimen.

**Table 2:** Predictors of achieving a CD4/CD8 ratio >1.

	Univariate			Multivariate 1			Multivariate 2		
	OR	95%CI	p	OR	95%CI	p	OR	95%CI	p
**Age (per 10 years)**	0.80	0.61–1.04	**0.09**	0.94	0.69–1.30	**0.72**	0.95	0.68–1.31	**0.73**
**Gender**									
Male	0.78	0.45–1.35	**0.37**	-	-		-	-	
Female	ref	-		-	-		-	-	
**Overweight BMI (**kg/m^2^ )*			**0.96**			**0.26**			**0.11**
BMI < 25	ref	-		ref	-		ref	-	
BMI > = 25	0.91	0.47–1.75		0.58	0.25–1.37		0.50	0.21–1.21	
Missing value	1.0	0.34–2.95		0.38	0.08–1.71		0.23	0.05–1.22	
**HCV coinfection**									
No	ref	-	**0.84**	ref	-	**0.21**	ref	-	**0.20**
Yes	0.94	0.51–1.74		1.89	0.70–5.12		1.90	0.71–5.10	
**AIDS-defining condition**									
No	ref	-	**0.012**	ref	-	**0.24**	ref	-	**0.22**
Yes	0.35	0.16–0.79		0.55	0.20–1.51		0.53	0.19–1.46	
**Mode of contamination**									
Heterosexual	ref	-	**0.076**	ref	-	**0.43**	ref	-	**0.27**
Homo-bisexual	0.62	0.34–1.13		0.53	0.25–1.16		0.47	0.21–1.02	
IV drug user	0.39	0.15–1.03		0.58	0.12–2.87		0.74	0.15–3.59	
Other	1.59	0.59–4.53		0.93	0.23–3.72		1.12	0.28–4.44	
**Calendar year**			**0.83**			**0.38**			**0.17**
≤ 2005	0.94	0.50–1.76		0.63	0.23–1.76		0.53	0.21–1.32	
> 2005	ref	-		ref	-		ref	-	
**Diagnosis-to-treatment delay** (years)	0.96	0.92–0.99	**0.027**	0.94	0.88–1.00	**0.04**	0.92	0.86–0.98	**0.010**
**Baseline CD4 count** (for 50 cells/mm^3^ increase)	1.13	1.08–1.19	**<0.0001**	0.99	0.89–1.10	**0.84**	1.00	0.90–1.11	**0.94**
**Nadir CD4** (for 50 cells/mm^3^ increase)	1.18	1.11–1.26	**<0.0001**	-	-		-	-	
**Baseline CD8 count** (for 50 cells/mm^3^ increase)	0.98	0.96–1.01	**0.21**	1.03	0.99–1.08	**0.15**	1.04	0.99–1.08	**0.14**
**Baseline ratio** (for 0.1 increase)	1.76	1.54–2.02	**<0.0001**	2.40	1.82–3.17	**<0.0001**	2.50	1.88–3.32	**<0.0001**
**Viral Load**			**0.20**			**0.0002**			**0.0001**
< = 500	ref	-		ref	-		ref	-	
500–30000	0.64	0.29–1.41		2.57	0.87–7.57		2.73	0.94–7.92	
>30000	1.20	0.67–2.16		10.8	3.75–31.3		14.4	4.74–43.6	
Missing value	0.21	0.028–1.60		1.56	0.17–14.1		1.78	0.19–16.6	
**Dyslipidaemia**						**0.07**			**0.04**
No	ref	-	**0.02**	ref	-		ref	-	
Yes	0.23	0.07–0.79		0.23	0.05–0.96		0.20	0.05–0.91	
Missing	0.54	0.30–0.96		0.53	0.25–1.11		0.45	0.21–0.96	
**Treatment (1)**			**0.38**			**0.29**			
NRTI+PI and PI only ± II ± EI	ref	-		ref	-		-	-	
NRTI+NNRTI ± II ± EI	0.83	0.45–1.52		0.77	0.35–1.69		-	-	
≥ 3 NRTI ± II ± EI	0.39	0.12–1.32		0.44	0.09–2.13		-	-	
Other: PI + NNRTI ± NRTI ± II ± EI	1.29	0.51–3.30		2.32	0.65–8.32		-	-	
**Treatment (2)**			**0.014**						**0.0003**
Non INSTI-containing regimens	ref	-		-	-		ref	-	
INSTI-containing regimens*	2.58	1.21–5.52		-	-		7.67	2.54–23.2	

Abbreviations—OR, odd-ratio; 95%CI, 95% confidence interval; p, p-value; BMI, body mass index; NRTI, nucleos(t)ide reverse transcriptase inhibitor; NNRTI, non-nucleotide reverse transcriptase inhibitor; PI, protease inhibitor; II, integrase inhibitor; EI, entry inhibitor.

Multivariate model 1 considered cART treatment as a 4 classes variable, multivariable model 2 considered cART treatment as binary variable II containing regimen or not.

In multivariate analysis, as planned, we fitted two models, each with a different coding for cART, adjusted on parameters known to be associated with normalization or significantly associated with it in univariate analysis. Gender was removed from these models as it was not independently associated with normalization and not reported to be so in the literature. Finally, our definitive multivariate models are presented in Table 2. In both models, CD4/CD8 ratio normalization was associated with shorter diagnosis-to-treatment delay, and higher baseline ratio and viral load. After adjustment, baseline dyslipidaemia still independently associated with the occurrence of CD4/CD8 ratio normalization [OR, 0.23 (0.07–0.79)]. There was no evidence of any influence of the type of cART regimen on ratio normalization when coded as PI, NNRTI or NRTI based regimen. However, when considering INSTI-containing regimen alone, it was strongly associated with normalization [OR, 7.67 (2.54–23.2)].

In sensitivity analysis, using a lower threshold of 0.8 for CD4/CD8 ratio normalization did not change the multivariate models results.

## Discussion

Few studies have investigated the association between cART regimens and CD4/CD8 ratio normalization and, to our knowledge, none have specifically looked after the integrase-inhibitors class.

In this population of naïve patients initiating treatment, we found that when stratifying first line cART as the main used regimens (PI-based (reference), NNRTI- based, NRTI alone, or other regimens) there was no association with ratio normalization in the first year. Similarly, in a large cohort of patients initiating cART, Leung et al did not found such an association [14]. In line with these results, no association was assessed between cART and the normalization of immune parameters defined as a composite outcome grouping CD4 cell count >500/mm^3^ plus % CD4 cell >29% plus CD4/CD8 ratio >1 in 352 patients commencing cART and achieving sustained undetectable viral load [7]. Conversely, in a recent study on more than 3000 HIV-patients initiating cART and reaching undetectable viral load, Mussini et al report an association between AZT/3TC or ddI/d4T use and a lower rate of ratio normalization, using Tenofovir/FTC combination as a reference [13]. No association was found with PI-class or NNRTI-class except in a sensitivity analysis where follow-up was censored at second viral load over 200 copies/ml (instead of first value over 80 copies/ml in main analysis) [13]. PI use was then associated with a lower rate of normalization compare to NNRTI. However, they used ART treatment as a time varying variable in their study whereas we intended to assess the impact of the first line regimen in an ITT principle.

According to our predefined analysis plan we also investigated the specific relation between INSTI-containing regimen use and CD4/CD8 ratio normalization. In our population, initiating ARV treatment with an INSTI-containing regimen was a strong independent predictor of ratio normalization within the first year of treatment. This association could be the result of the effect of raltegravir on immune activation and inflammation that has been suggested in several switch studies from NNRTI- or PI-based regimen to raltegravir [15–17, 28] or intensification studies [18, 21, 23]. However, the effect on specific markers of activation or inflammation was sometimes inconsistent across the trials [19]. Nevertheless, in a small prospective cohort study of 25 patients a specific improvement of the CD4/CD8 ratio with raltegravir was already reported after switching from NNRTI- or PI-based regimen [22]. Lastly, in 2 recent studies, ratio normalization was associated with a cART initiation in the more recent years [13, 14] potentially traducing the impact of these new drugs in first-line regimens.

Recent Guidelines for HIV treatment [29, 30] recommend treating patients as early as possible in the course of HIV infection, regardless of symptomatology, CD4 count or VL. Only for asymptomatic patients with CD4 count >500 cells/mm^3^ this still debated and results of the START study are awaited [31]. Indeed, the period of immune restoration after ARV treatment initiation (which still occurs at a low CD4 count as patients are often diagnosed late) is at high risk of morbidity and should be shorten as much as possible. Also, inverted CD4/CD8 ratio has been shown to be an independent factor of morbidity in HIV-patients and can still low even in those with a controlled viral load and CD4 count recovery above 500 cells/mm^3^ with cART [9]. Though, in this context, the choice of a cART regimen, especially first-line regimen, is of crucial importance, taking into account VL control, CD4 count recovery as well as CD4/CD8 ratio normalization in a short delay.

We observed in our study a 10.9% rate of ratio normalization at one year. This rate seems slightly higher than what has been previously observed in other studies with rates of 4.4% [13] and around 5% [14] at one year. Differences in outcome evaluation or study population across the studies may, at least partly, account for this. Indeed, Mussini et al excluded from their study the patients who normalized their ratio between ART initiation and viral suppression and normalization was assessed after 2 consecutive ratio measurements above the predefined cut-off instead of one in our study [13, 14]. Interestingly we found, in multivariate models, that a higher baseline VL was associated with increased probability of ratio normalization as it has been previously reported [7]. As we included all the patients in our study irrespective of viral load and not only those who reached viral suppression after cART initiation, this may have played a role. The chosen cut-off for normalization, ≥1, similar to previous studies [7, 13], was lower than in the study by Leung *et al* [14] where patients had to reach a more conservative cut-off of 1.2 to achieve normalization. This may also explain the higher rate of normalization observed in our study. Nevertheless, normalization rates still quite low in all studies reflecting that complete restauration of the immunological functions in HIV patients is rare even in case of ART efficacy.

Dyslipidemia has been associated with increased levels of markers of inflammation, as well as thrombogenesis and endothelial dysfunction. As so, we hypothesized that it may be a predictor of ratio normalization. In multivariate analysis, after adjustment on known risk factors, dyslipidaemia was associated with an 80% reduction of the chance to achieve normalization in the year after treatment initiation. This may be in favour of an independent role in chronic inflammation and though on immune restauration.

There are limitations to our study. As only the raltegravir was in routine use by the time we collected data, it is the only drug we observed in the INSTI-class in this study, potentially limiting our conclusions at the drug-class level. Indeed, French regulations’ approval for the dolutegravir and the elvitegravir are posterior to the end of the data collection for this study. Nevertheless we should also consider that only 8% (45 patients) of our study population were receiving raltegravir as part of their first-line regimen, potentially limiting the power of our analysis. However, the OR associated with INSTI use is highly significant [7.67 (2.54–23.2), p = 0.0003] and a larger population would have most probably affected the CI95% of the OR but not the direction of the association even if we cannot definitively rule-out the possibility of an allocation bias secondary to a small selected subgroup of patients receiving raltegravir. This has been, at least partly, taken into account through the multivariate analysis with adjustment on variables associated with ratio normalization. Longer follow-up could have allow to observe higher rate of normalization as studies have found a median delay to ratio normalization ranging from 3 to 10.1 years [13, 14]. However, longer follow-up would have mean more lost to follow-up patients and the risk of powerless analysis as well as a more complex interpretation of the results as the number of ARV treatment lines increases with time. Indeed we did not censor our data at the first ARV change but this bias is limited when we censor the follow-up at one year. Furthermore our question was centered on the short term normalization with first-line regimen.

Our findings suggest an association between initiation of an INSTI-containing regimen and CD4/CD8 ratio normalization at one year. This result is assessed on a population unselected on viral load, naïve of ARV, so effect of INSTI-class is not confounded by prior ARV treatment, in “real life condition”. However this should be confirmed on a larger naïve population, receiving all INSTI drugs, with an extended follow-up to draw firm conclusions. Our results support the recent update of the “Guidelines for the Use of Antiretroviral Agents in HIV-1-Infected Adults and Adolescents” [29] that largely recommends use of INSTI-containing regimen for naïve patients. Indeed, as CD4/CD8 ratio is considered as an independent predictor for morbidity in HIV, association of INSTI and normalization is another argument for their use as first-line regimen.
